# Association of physical activity pattern and risk of Parkinson’s disease

**DOI:** 10.1038/s41746-024-01135-3

**Published:** 2024-05-23

**Authors:** Fabin Lin, Yixiang Lin, Lina Chen, Tingting Huang, Tianxin Lin, Jiarui He, Xiaoyang Lu, Xiaochun Chen, Yingqing Wang, Qinyong Ye, Guoen Cai

**Affiliations:** 1 Department of Neurology, Center for Cognitive Neurology, Institute of Clinical Neurology, Fujian Medical University Union Hospital, 29 Xinquan Road, Fuzhou, 350001 China; 2 Fujian Institute of Geriatrics, Fujian Medical University Union Hospital, 29 Xinquan Road, Fuzhou, 350001 China; 3 Fujian Key Laboratory of Molecular Neurology, Fujian Medical University, 88 Jiaotong Road, Fuzhou, 350001 China; 4 Department of Neurosurgery, Fujian Medical University Union Hospital, 29 Xinquan Road, Fuzhou, 350001 China

**Keywords:** Risk factors, Parkinson's disease

## Abstract

Increasing evidence suggests an association between exercise duration and Parkinson’s disease. However, no high-quality prospective evidence exists confirming whether differences exist between the two modes of exercise, weekend warrior and equal distribution of exercise duration, and Parkinson’s risk. Hence, this study aimed to explore the association between different exercise patterns and Parkinson’s risk using exercise data from the UK Biobank. The study analyzed data from 89,400 UK Biobank participants without Parkinson’s disease. Exercise data were collected using the Axivity AX3 wrist-worn triaxial accelerometer. Participants were categorized into three groups: inactive, regularly active, and engaged in the weekend warrior (WW) pattern. The relationship between these exercise patterns and Parkinson’s risk was assessed using a multifactorial Cox model. During a mean follow-up of 12.32 years, 329 individuals developed Parkinson’s disease. In a multifactorial Cox model, using the World Health Organization–recommended threshold of 150 min of moderate-to-vigorous physical activity per week, both the active WW group [hazard ratio (HR) = 0.58; 95% confidence interval (CI) = 0.43–0.78; *P* < 0.001] and the active regular group (HR = 0.44; 95% CI = 0.34–0.57; *P* < 0.001) exhibited a lower risk of developing Parkinson’s disease compared with the inactive group. Further, no statistically significant difference was observed between the active WW and the active regular groups (HR = 0.77; 95% CI = 0.56–1.05; *P* = 0.099). In conclusion, in this cohort study, both the WW exercise pattern and an equal distribution of exercise hours were equally effective in reducing Parkinson’s risk.

## Introduction

Parkinson’s disease (PD) is a neurodegenerative disorder characterized by muscle tonus, slow movements, postural instability, and resting tremors. PD affects more than 4.1 million people globally, mostly individuals older than 50 years. The number of affected individuals is expected to reach 8.7–9.3 million by 2030^1^. PD is a multifactorial disease influenced by genetic predisposition, environmental factors, and lifestyle choices, including daily exercise^2,3^. Given the significant burden of PD, identifying risk factors and developing preventive interventions are crucial public health priorities.

Increasing evidence indicates that exercise not only reduces the risk of PD but also holds significant implications for relieving symptoms in patients with PD and preventing depression associated with the disease^4–6^. The World Health Organization guidelines recommend 150 min of moderate-to-vigorous physical exercise (MVPA) each week^7^. Several cohort studies have shown that individuals who complete the majority of their weekly exercise in 1–2 days (weekend warriors) experience similar benefits in terms of reducing cardiovascular disease, depression, and various other disorders compared with those who evenly distribute their exercise time throughout the week^8–10^. However, no research has been conducted to determine which pattern of exercise is more effective in lowering the risk of PD and whether the number of hours of activity per week is evenly distributed or concentrated on 1 or 2 days. Consequently, participants were categorized into active and inactive groups, with the active group further divided into an active regular group and an active WW group based on the distribution of exercise time^8^.

The primary objective of this study was to investigate the association between two different exercise patterns and the incidence of PD in a cohort from the UK Biobank. The study compared individuals who completed most of their weekly exercise in 1–2 days (WWs) with those who evenly distributed their weekly exercise time (active regular).

## Results

### Characteristics of participants

This study included 89,400 individuals, with a mean follow-up period of 12.32 years. During the follow-up period, 329 participants developed PD. The MVPA data as defined by the Axivity AX3 wrist-worn triaxial accelerometer. The baseline table indicates that the active WW group had a higher proportion of men, higher income levels, increased education levels, lower Thomson Deprivation Index (TDI), fewer recent smokers, more recent drinkers, fewer patients with diabetes, and a higher weekly duration of MVPA compared with the inactive group. Participants in the active WW group shared identical study characteristics with those in the active regular group, except for a lower weekly MVPA duration in the active WW group (Table 1).

**Table 1:** Sample characteristics of participants in an accelerometer-derived physical activity study

Variables	Characteristics of overall participants				
	Overall	Active regular^a^	Inactive^a^	Active WW^a^	*P* value^b^
No.	89,400	20,381	31,778	37,241	
Age(mean (SD))	56.05 (7.84)	54.94 (7.87)	56.79 (7.86)	56.04 (7.74)	<0.001
Sex(%)					
Female	50,308 (56.3)	10,326 (50.7)	20,843 (65.6)	19,139 (51.4)	<0.001
Male	39,092 (43.7)	10,055 (49.3)	10,935 (34.4)	18,102 (48.6)	
BMI(mean (SD))	26.70 (4.50)	25.79 (4.00)	27.93 (5.10)	26.14 (3.95)	<0.001
TDI (mean (SD))	−1.72 (2.81)	−1.29 (3.00)	−1.77 (2.78)	−1.92 (2.69)	<0.001
Average gross household income before taxes(%)					
18,000 to 100,000	63,476 (71.0)	14,664 (71.9)	21,735 (68.4)	27,077 (72.7)	<0.001
Greater than 100,000	6160 (6.9)	1771 (8.7)	1531 (4.8)	2858 (7.7)	
Less than 18,000	11,592 (13.0)	2379 (11.7)	5041 (15.9)	4172 (11.2)	
Unknow	8172 (9.1)	1567 (7.7)	3471 (10.9)	3134 (8.4)	
Healthdietscore (%)					
0	1545 (1.7)	341 (1.7)	600 (1.9)	604 (1.6)	<0.001
1	8132 (9.1)	1780 (8.7)	3105 (9.8)	3247 (8.7)	
2	17,754 (19.9)	3749 (18.4)	6614 (20.8)	7391 (19.8)	
3	24,909 (27.9)	5640 (27.7)	8815 (27.7)	10,454 (28.1)	
4	23,881 (26.7)	5623 (27.6)	8198 (25.8)	10,060 (27.0)	
5	13,179 (14.7)	3248 (15.9)	4446 (14.0)	5485 (14.7)	
Race (%)					
Asia	916 (1.0)	206 (1.0)	409 (1.3)	301 (0.8)	<0.001
Black	255 (0.3)	65 (0.3)	116 (0.4)	74 (0.2)	
Other	4784 (5.4)	1339 (6.6)	1574 (5.0)	1871 (5.0)	
White	83,445 (93.3)	18,771 (92.1)	29,679 (93.4)	34,995 (94.0)	
MVPA (mean (SD))	282.28 (251.60)	495.45 (292.59)	68.44 (47.69)	348.08 (186.97)	<0.001
smoke (%)					
No	83,243 (93.1)	19,111 (93.8)	29,016 (91.3)	35,116 (94.3)	<0.001
Only occasionally	2009 (2.2)	504 (2.5)	699 (2.2)	806 (2.2)	
Yes, on most or all days	4148 (4.6)	766 (3.8)	2063 (6.5)	1319 (3.5)	
alcohol (%)					
Current	84,485 (94.5)	19,360 (95.0)	29,524 (92.9)	35,601 (95.6)	<0.001
Never	2503 (2.8)	488 (2.4)	1205 (3.8)	810 (2.2)	
Previous	2412 (2.7)	533 (2.6)	1049 (3.3)	830 (2.2)	
Hypertension (%)					
Elevated	12,054 (13.5)	2880 (14.1)	3991 (12.6)	5183 (13.9)	<0.001
Normal	15,137 (16.9)	3823 (18.8)	4918 (15.5)	6396 (17.2)	
stage1	24,663 (27.6)	5754 (28.2)	8686 (27.3)	10,223 (27.5)	
stage2	37,546 (42.0)	7924 (38.9)	14,183 (44.6)	15,439 (41.5)	
Diabetes (%)					
No	85,856 (96.0)	19,781 (97.1)	29,891 (94.1)	36,184 (97.2)	<0.001
Yes	3544 (4.0)	600 (2.9)	1887 (5.9)	1057 (2.8)	
Family history of Parkinson’s disease (%)					
No	85,303 (95.4)	19,409 (95.2)	30,341 (95.5)	35,553 (95.5)	0.35
Yes	4097 (4.6)	972 (4.8)	1437 (4.5)	1688 (4.5)	
education (%)					
Any school degree	11,926 (13.3)	2681 (13.2)	4303 (13.5)	4942 (13.3)	<0.001
College or University degree	39,048 (43.7)	10,204 (50.1)	11,349 (35.7)	17,495 (47.0)	
Other	34,773 (38.9)	6736 (33.1)	14,561 (45.8)	13,476 (36.2)	
Vocational	3653 (4.1)	760 (3.7)	1565 (4.9)	1328 (3.6)	

*BMI* Body Mass Index, *TDI* Townsend Deprivation Index, *VPA* moderate to vigorous physical activity

^a^Threshold for MVPA is 150 minutes.

^b^Analysis of variance or x^2^ test where appropriate

**Fig. 1:**
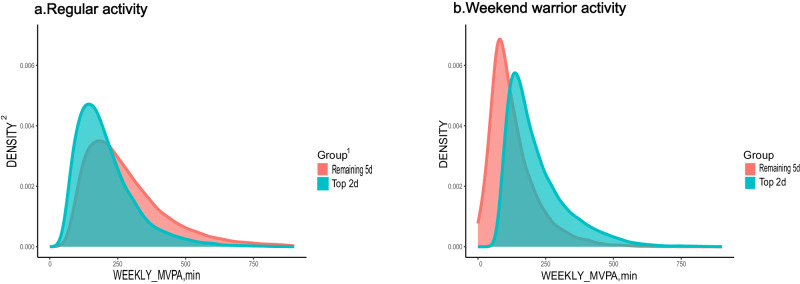
Distribution of moderate to vigorous physical activity (MVPA) on top 2 days vs remaining 5 days among active individuals using guideline-based activity threshold of 150 min or more of MVPA per week. **a** Regular activity and **b** Weekend warrior activity. ^1^Top 2d means the sum of the two days with the most MVPA hours in a week; Remaining 5d means the total MVPA hours for the remaining 5 days. ^2^the density of the y-axis represents the probability density. The y-axis reflects the relative numbers of people corresponding to each MVPA minutes.

Additionally, the density plot depicting the distribution of MVPA exercise hours between the active WW and active regular groups revealed that the two most active days in the active WW group had a higher concentration of MVPA exercise hours compared with the other 5 days. In contrast, the exercise hours in the active regular group were more evenly distributed (Fig. 1).

### Association between different exercise patterns and the risk of PD

**Fig. 2:**
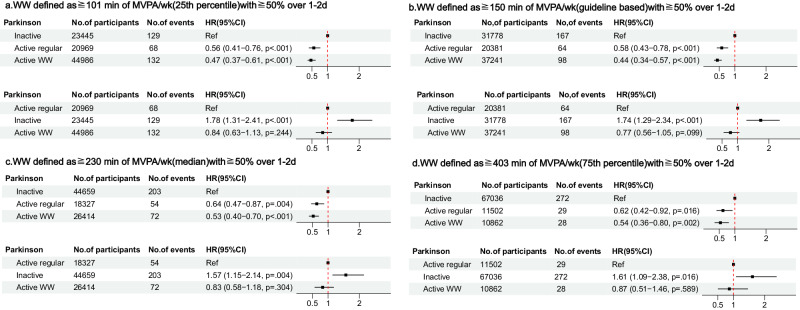
Association between physical activity patterns and Parkinson’s disease. **a** WW defined as ≧101 min of MVPA/wk (25th percentile)with ≧50% over 1–2d, **b** WW defined as ≧150 min of MVPA/wk(guideline based) with ≧50% over 1–2d, **c** WW defined as ≧230 min of MVPA/wk (median)with ≧50% over 1–2d and **d** WW defined as ≧403 min of MVPA/wk (75th percentile) with ≧50% over 1–2d.

Regarding the relationship between the risk of PD and different exercise modes, we found that when the MVPA threshold was set at 150 minutes, both the active WW group [hazard ratio (HR) = 0.58; 95% confidence interval (CI) = 0.43–0.78; *P* < 0.001] and the active regular group (HR = 0.44; 95% CI = 0.34–0.57; *P* < 0.001) exhibited a lower risk of developing PD compared with the inactive group. We employed thresholds at 25%, 50%, and 75% of participants’ weekly MVPA time to ensure the stability of the results. At the 101-min threshold, the risk of PD was lower in both the active WW group (HR = 0.56; 95% CI = 0.41–0.76; *P* < 0.001) and the active regular group (HR = 0.47; 95% CI = 0.37–0.61; *P* < 0.001) than in the inactive group. At a median threshold of 230 min of MVPA per week, both the active WW group (HR = 0.64; 95% CI = 0.47–0.87; *P* = 0.004) and the active regular group (HR = 0.53; 95% CI = 0.40–0.70; *P* < 0.001) exhibited a lower risk than the inactive group. Finally, at the 403-min threshold, both the active WW group (HR = 0.62; 95% CI = 0.42–0.92; *P* = 0.016) and the active regular group (HR = 0.54; 95% CI = 0.36–0.80; *P* = 0.002) had a lower risk of PD compared with the inactive group (Fig. 2).

When using the active regular group as the reference group and setting the MVPA threshold at 150 min, the inactive group (HR = 1.74; 95% CI = 1.29–2.34; *P* < 0.001) exhibited a higher risk of PD than the active regular group, whereas no statistically significant difference was observed in the active WW group compared with the active regular group (HR = 0.77; 95% CI = 0.56–1.05; *P* = 0.099). At the 101-min threshold, the inactive group (HR = 1.78; 95% CI = 1.31–2.41; *P* < 0.001) showed a higher risk of PD compared with the active regular group. However, no statistically significant difference was observed in the active WW group compared with the active regular group (HR = 0.84; 95% CI = 0.63–1.13; *P* = 0.224). At the 230-min threshold, the inactive group (HR = 1.57; 95% CI = 1.15–2.14; *P* = 0.004) had a higher risk of PD than the active regular group. However, no statistically significant difference was observed in the active WW group compared with the active regular group (HR = 0.83; 95% CI = 0.58–1.18; *P* = 0.304). Finally, at the 403-min threshold, the inactive group (HR = 1.61; 95% CI = 1.09–2.38; *P* = 0.016) exhibited a higher risk of PD than the active regular group. However, no statistically significant difference was observed in the active WW group compared with the active regular group (HR = 0.87; 95% CI = 0.51–1.46; *P* = 0.589).

### Sensitivity analysis

Two models were employed while plotting the survival curve analysis using the Kaplan–Meier method. In the first model, participants were categorized into three groups: the inactive group, the active regular group, and the active WW group, with a weekly MVPA duration of 150 min as the threshold. Significant differences were observed between the Kaplan–Meier curves of the inactive group and either the active regular group (*P* < 0.001) or the active WW group (*P* < 0.001). However, no statistically significant difference was observed between the active regular and active WW groups (*P* = 0.2675) (Supplementary Figure 2). In the alternative model, where the threshold was removed, participants were directly categorized into the active regular and active WW groups, and no statistically significant difference was observed between the Kaplan–Meier curves of the active regular and active WW groups (*P* = 0.24) (Supplementary Figure 3). When individuals were categorized into four groups based on MVPA duration without any threshold, no significant difference was found in the probability of PD onset between the active regular and active WW groups (Supplementary Table 2). In addition, after excluding patients with PD of less than 2 years duration, the findings remained consistent with the previous analysis (Supplementary Figure 1). Subgroup analyses were conducted using sex (*P* for interaction = 0.1145), blood pressure status (*P* for interaction = 0.3475), drinking status (*P* for interaction = 0.4532), diabetes (*P* for interaction = 0.06578), and family history of PD (*P* for interaction = 0.3475). However, no covariates showed significant interactions with exercise patterns (Supplementary Table 1). Utilizing 75% of the total MVPA achieved in 1–2 days as a threshold to distinguish between the active regular group and active WW group yielded results similar to using 50% as a threshold (Supplementary Figure 4). No significant association was observed between the distribution of exercise intensity and the risk of PD (Supplementary Table 3), and no significant nonlinear associations were found in the RCS association (*P* for nonlinear = 0.7068)(Supplementary Figure 5).

## Discussion

This large prospective cohort study demonstrated that both exercise patterns, whether concentrated on weekends or evenly distributed throughout the week, were significantly associated with a reduced risk of developing PD. The study highlighted that the choice between being a WW or maintaining an even distribution of exercise time was equally effective in preventing the onset of PD. In addition, we conducted subgroup analyses examining five covariates: sex, drinking status, blood pressure status, diabetes, and family history of patients with PD. The results showed that these covariates did not significantly interact with exercise. This suggested that the conclusions drawn from the study were more broadly applicable to diverse populations, extending beyond specific characteristics.

Several studies examined the relationship between exercise and PD^11–13^. A large prospective cohort study revealed that higher levels of exercise could reduce the risk of PD in men. However, no significant correlation was found between exercise levels and the risk of PD in women^11^. A large-scale meta-analysis also supports these findings^12^. In contrast, another large cohort study involving older adults in the United States found an association between higher levels of moderate-to-vigorous exercise and a lower risk of PD in both men and women^13^. These results were consistent with our findings, indicating a lower risk of PD development in the active group than in the inactive group.

However, most existing studies focused on assessing the protective effect of exercise duration on the risk of PD. High-quality evidence on the relationship between exercise frequency and the prevention of PD is scarce. Although previous studies have reported that WWs may experience similar risk reduction for cardiovascular and psychiatric diseases as those with a consistent exercise schedule^8,10^, such information is not yet available for PD. This study represented the large-scale cohort investigation exploring the association between exercise patterns and the risk of developing PD. Our findings demonstrated that both modes of exercise, intensive weekend workouts and evenly distributed exercise time, had the same significant effect in preventing the onset of PD. This suggested that the duration of exercise might have a larger impact in terms of reducing the risk of PD compared with the frequency of exercise.

In sensitivity analyses, we conducted subgroup analyses of participants to investigate potential differences in the associations between exercise patterns and PD across populations with different characteristics. We collectively considered five covariates, including sex, drinking status, blood pressure status, diabetes mellitus, and family history of PD, for these subgroup analyses. The results did not reveal any significant interactions between these covariates and movement patterns. Therefore, it was assumed that the association between movement patterns and PD was consistent across individuals with different characteristics, suggesting that the conclusions of this study could be applied to populations with different baseline characteristics.

This study holds significant implications for understanding how the allocation of time to physical activity can be used in preventing PD. Considering that both exercise patterns have a similar effect in reducing the risk of PD, individuals can choose either option based on their own habits in daily life.

However, certain drawbacks to the WW model of the movement also exist. Evidence indicates that WWs are usually people who lack exercise during the weekdays. As a result of this reduced weekly exercise, they usually focus on their entire week’s exercise within 1–2 exercise sessions. The lack of proper conditioning and supplementary training for WWs makes them more susceptible to skeletal muscle injuries compared with individuals who maintain a consistent exercise routine throughout the week. However, this heightened risk of musculoskeletal disorders remains comparable to those observed in individuals following a more evenly distributed exercise regimen^14^.

This study had some limitations. (1) The UK Biobank only captured one week of MVPA exercise time for each participant and did not take multiple measurements. According to the Hawthorne effect, if participants are only monitored for 1 week, it is possible that their behavioral patterns may be influenced only for that particular week, and may not accurately reflect their true activity patterns. (2) The MVPA exercise hours in the UK Biobank were measured using an Axivity AX3 wrist-worn triaxial accelerometer. Although this device has been applied for a wide range of exercise modes, exercise data cannot be accurately collected for certain activities, leading to incomplete coverage of all exercise modes. (3) Given that most of the participants in the UK Biobank study were of white ethnicity, with a small representation from other racial groups, further research and longer monitoring in more diverse populations and geographic regions are needed to confirm these results and increase their generalizability. (4) As motion data in this study were all measured using a wrist-watch accelerometer, further investigations are needed to determine whether the motion data obtained by other means aligned with those recorded by a wrist-worn accelerometer. (5) The limited number of PD cases might have hindered subgroup analyses for some covariates, such as ethnicity, smoking status, education level, and healthy diet scores.

## Methods

### Study design and participants

The UK Biobank is a large-scale cohort study conducted between 2006 and 2010, encompassing about 500,000 individuals aged 37–73 years. The baseline data were collected at 22 study locations in England, Scotland, and Wales using touchscreen surveys, interviews, physical and functional assessments, as well as genetic and biological procedures^15^. The study was approved by the North West Multi-centre Research Ethics Committee, the National Information Management Board, and the UK government. The ethical considerations for research involving individuals were thoroughly reviewed, and participants provided written informed consent.

**Fig. 3:**
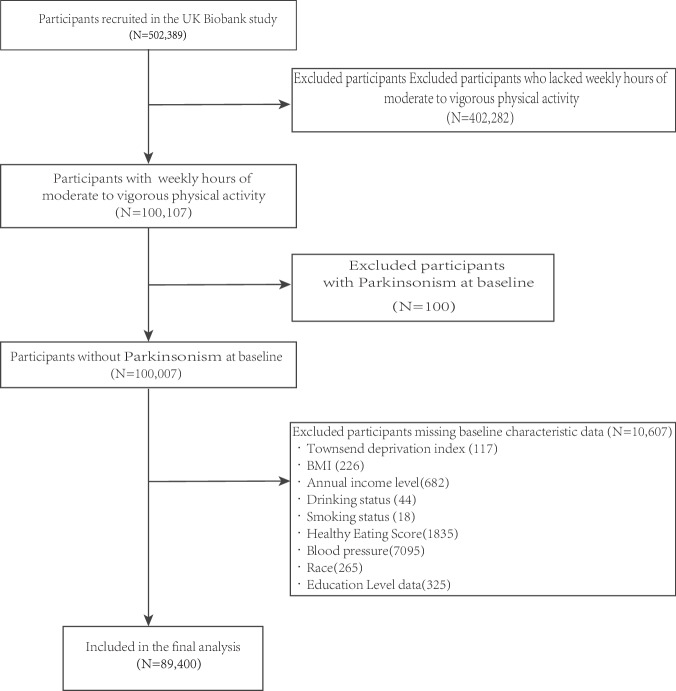
Selection of study participants in the UK Biobank. The diagram provides a schematic illustration for the participant inclusion process in our study.

Initially, this cohort study recruited 502,389 individuals from the UK Biobank. Further, 402,282 participants lacking information on daily and weekly MVPA exercise hours were excluded. Additionally, 100 participants with pre-existing PD were excluded. Finally, 10,607 participants with missing covariates, such as their health status and healthy eating scores, were excluded. Hence, the final study cohort comprised 89,400 individuals (Fig. 3).

### Moderate-to-vigorous physical activity

This study assessed the weekly MVPA duration and the daily MVPA duration using data from the UK Biobank. Participants in the UK Biobank used an Axivity AX3 wrist-worn triaxial accelerometer to evaluate their MVPA duration^16^. A previous study validated the reliability of this instrument for recording exercise duration^17^. Further, the Axivity AX3 wrist-worn triaxial accelerometer demonstrated comparable results to GENEActiv accelerometers used in other large-scale cohort studies^18–20^. The Axivity device was configured to capture three-axis acceleration data at 100 Hz during a 7-day period, with a dynamic range of ±8 g. The acceleration signal was calibrated to local gravity by the UK Biobank, ensuring a standard error for the *x*/*y*/*z* axis of less than 13.0 mg. Additionally, the Axivity AX3 wrist-worn triaxial accelerometer could recognize various modes of movement (cycling, running, and swimming) through machine deep learning. The UK Biobank considers nonwearing time, defined as a period when the sensor does not collect exercise data for 60 min and more. The nonwearing time is excluded from the computational model, and only the daily wearing time is used in computing the participant’s average moderate-to-high intensity exercise.

This study categorized the participants into three groups based on the World Health Organization’s recommendation of 150 min of MVPA as a cutoff point and whether 50% of MVPA was concentrated on 1 or 2 days per week. The participants were categorized as follows: inactive group (MVPA < 150 min), active regular group (MVPA ≧150 min, with <50% of total MVPA achieved in 1–2 days), and active WW group (MVPA≧150 min, with≧50% of total MVPA achieved in 1–2 days).

### Definition of PD

The UK Biobank identifies specific diseases such as PD and dementia using a unique algorithm known as “algorithmically defined outcomes (ADOs)”^21^. The algorithm assesses whether a participant has a particular disease by analyzing data collected from the participant’s baseline information and admission records, including self-reported data on medical issues, procedures, drugs, or death registries obtained from hospital-related data. These specific disorders are categorized using the 9th and 10th editions of the International Classification of Diseases (ICD-9 and ICD-10). The reliability of the UK Biobank’s ADOs has been demonstrated in a cohort study of PD^22^, and ADOs have been used in numerous cohort studies related to PD^23,24^.

The primary endpoint of this study was the occurrence of PD, defined according to the ICD-9 and ICD-10.

### Assessment of covariates

The UK Biobank used a touchscreen questionnaire to collect participants’ information, including sex, age, ethnicity (Asian, Black, White, and Other), smoking status (never smoked, only occasionally, and yes – on most or all days), drinking status (never, ever, and now), education (university degree, any school degree, vocational degree, and other), income (below £18,000, between £18,000 and £100,000, above £100,000, and unaware of annual household pre-tax income), and family history of PD (yes or no). The TDI is based on the postcode of the participant’s place of residence, and its calculation considers aggregated data on self-reported car ownership, unemployment, homeownership, and household overcrowding. The Healthy Diet Score is determined by the participant’s weekly consumption of vegetables, fruits, fish, meat, and so on, on a scale from 0 to 5, where higher scores indicate a healthier diet^25^. Diabetes status (yes or no) was established based on the participant’s self-reported doctor’s diagnosis and the use of antidiabetic medication. The blood pressure was calculated by averaging two seated measures taken at baseline using an Omron HEM-7015IT digital blood pressure monitor. Blood pressure was classified into four groups based on the 2017 American Heart Association guidelines^26^: normal (systolic and diastolic blood pressure of 120 and 80 mm Hg, respectively); elevated (systolic and diastolic blood pressure of 120–129 and 80 mm Hg, respectively); stage I hypertension (systolic and diastolic blood pressure of 130–139 and 80–89 mm Hg, respectively); and stage II hypertension (systolic and diastolic blood pressure of 130–139 and ≥90 mm Hg, respectively). In addition, when participants joined the UK Biobank study, a trained professional measured their height and weight to calculate their body mass index (BMI) using the BMI formula.

### Data analysis

This study used a multifactorial Cox model, adjusting for covariates such as age, sex, BMI, ethnicity, self-reported healthy eating score, TDI, annual income level, smoking, alcohol consumption, diabetes mellitus, hypertension, family history of PD, and the level of education, to investigate the association between exercise patterns and the risk of PD. The multivariate Cox model analysis included two reference groups: the inactive group and the active regular group. Besides using the World Health Organization’s recommended threshold of 150 min of MVPA per week to distinguish between the inactive and active groups, the thresholds of 25%, 50%, and 75% of the participants’ MVPA durations were also considered for analysis to ensure model stability.

Several sensitivity analyses were conducted to ensure the stability of the results: (1) The multifactorial Cox model was re-evaluated after excluding patients with PD with a disease duration of less than 2 years. (2) Kaplan–Meier survival curves were plotted for the three exercise patterns to assess potential differences in the risk of morbidity among the three groups. Additionally, the MVPA threshold was eliminated, and patients were categorized into two groups immediately: the active regular and active WW groups, with Kaplan–Meier survival curves drawn for these two groups. (3) Within each MVPA group, the participants were further divided into four equal groups based on the length of MVPA. The multifactorial Cox model described earlier was then applied to examine a difference in the risk of developing PD between the active regular and active WW groups within each MVPA group. (4) Subgroup analyses were performed to assess potential interactions between covariates and exercise patterns among participants. (5) We also adjusted the thresholds to define the active WW and regular groups to evaluate the reliability of the results. We used 150 min of MVPA duration at baseline as the threshold to distinguish between the inactive and active groups. Additionally, we examined whether 75% of MVPA per week was concentrated within 1 or 2 days as the group thresholds to define the active regular and WW groups. (6) We used the TOP 2d Percentage of total MVPA hours for the week to assess the distribution of exercise intensity. After excluding the inactive group with less than 150 min of MVPA per week, we used Cox modeling to investigate the risk between TOP 2d Percentage of total MVPA hours for the week and PD. Furthermore, a restricted cubic spline was used to examine the nonlinear relationship.

All statistical analyses were performed with R software version 4.2.3. Two-sided *p* < 0.05 was considered statistically significant.

### Reporting summary

Further information on research design is available in the Nature Research Reporting Summary linked to this article.

### Supplementary information

Supplemental material

Reporting Summary

## Data Availability

Data obtained from the UK Biobank are available on application at www.ukbiobank.ac.uk/register-apply (94166).

## Code availability

All statistical analyses in this study were performed using R software version 4.2.3. The analytic methods, code, and study materials that support the findings of this study will be available from the corresponding authors on request.
