# Supplementary material for: Association of physical activity pattern and risk of Parkinson’s disease
Source: NPJ Digit Med. 2024 May 23;7:137. doi: 10.1038/s41746-024-01135-3 (PMC11116521; doi:10.1038/s41746-024-01135-3)
Supplement: Supplementary file 1 — Supplemental material [file 41746_2024_1135_MOESM1_ESM.pdf]

List of supplementary materials

Supplementary Figure 1. Association of Physical Activity Patterns with Parkinson's Disease (Removal of Parkinson's Patients with Less Than 2 Years of Disease)

Supplementary Figure 2. Parkinson's Survival Curves for Three Groups After Thresholding at the Guideline-Recommended 150 Minutes of MVPA Duration Per Week

Supplementary Figure 3. Parkinson's survival curves for both groups after cancellation of thresholds

Supplementary Figure 4. Association between physical activity patterns and Parkinson's disease

Supplementary Figure 5. Restricted cubic spline for testing the hypothesis of nonlinear correlation between TOP 2d Percentage of total MVPA hours for the week and the risk of PD.

Supplementary Table 1. Subgroup analysis of the association between participant movement patterns and Parkinson's disease

Supplementary Table 2. Parkinson's risk between exercise modes after threshold cancellation

Supplementary Table 3. The effect of distribution of exercise intensity on Parkinson's risk

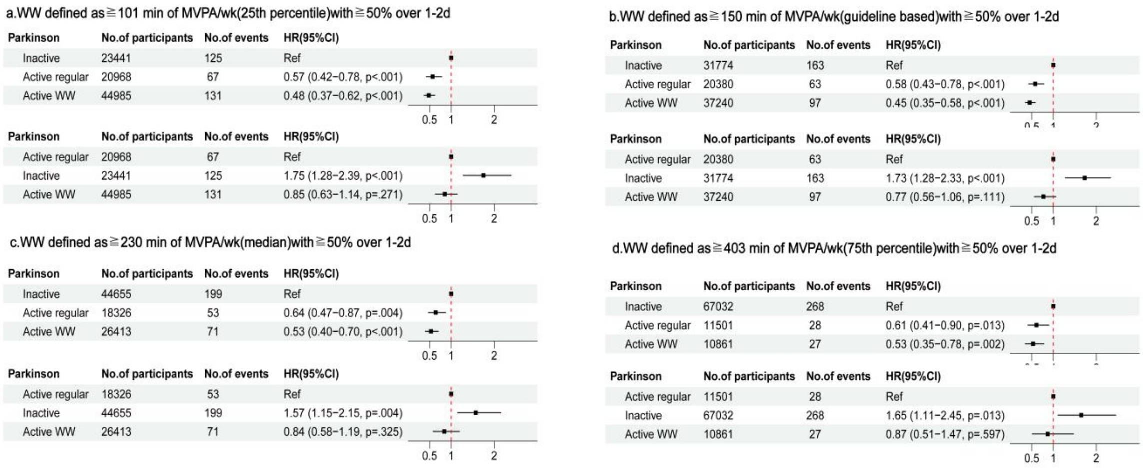

Supplementary Figure 1. Association of Physical Activity Patterns with Parkinson's Disease (Removal of Parkinson's Patients with Less Than 2 Years of Disease)

a.WW defined as≧101 min of MVPA/wk(25th percentile)with≧50% over 1-2d, **b.** WW defined as≧150 min of MVPA/wk(guideline based)with≧50% over 1-2d, **c.**WW defined as≧230 min of MVPA/wk(median)with≧50% over 1-2d and **d.** WW defined as≧403 min of MVPA/wk(75th percentile)with≧50% over 1-2d.

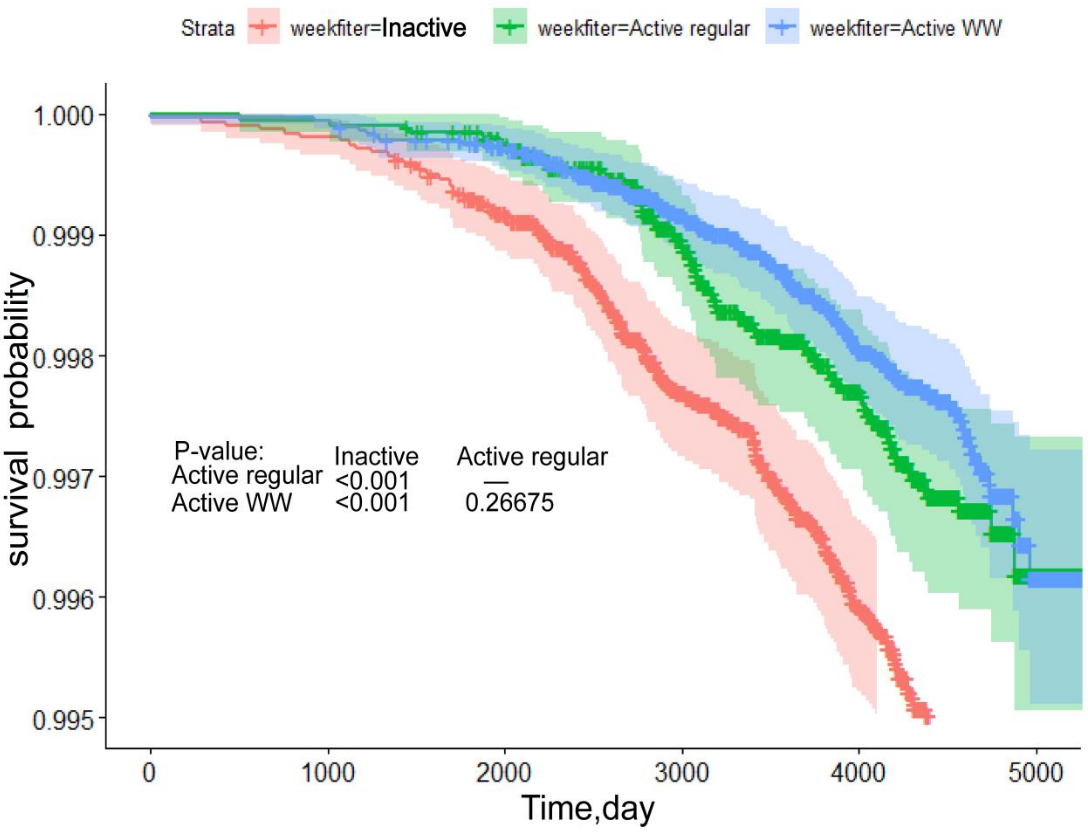

Supplementary Figure 2. Parkinson's Survival Curves for Three Groups After Thresholding at the Guideline-Recommended 150 Minutes of MVPA Duration Per Week

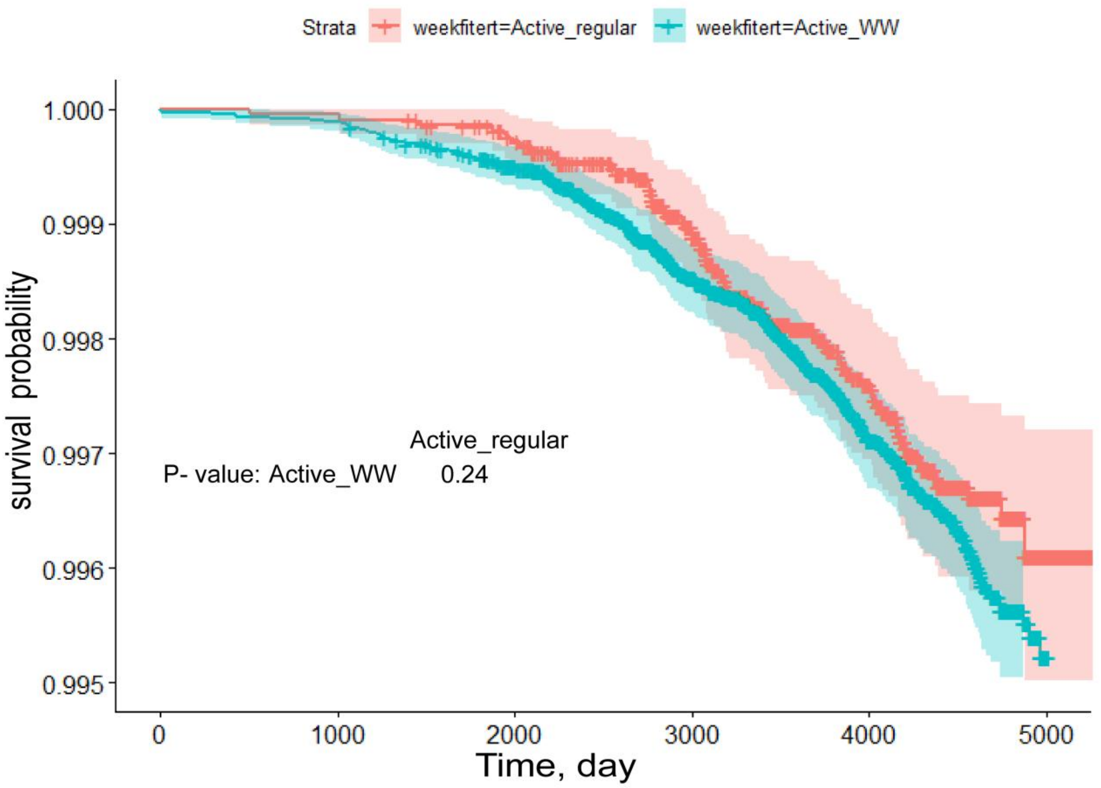

Supplementary Figure 3. Parkinson's survival curves for both groups after cancellation of thresholds

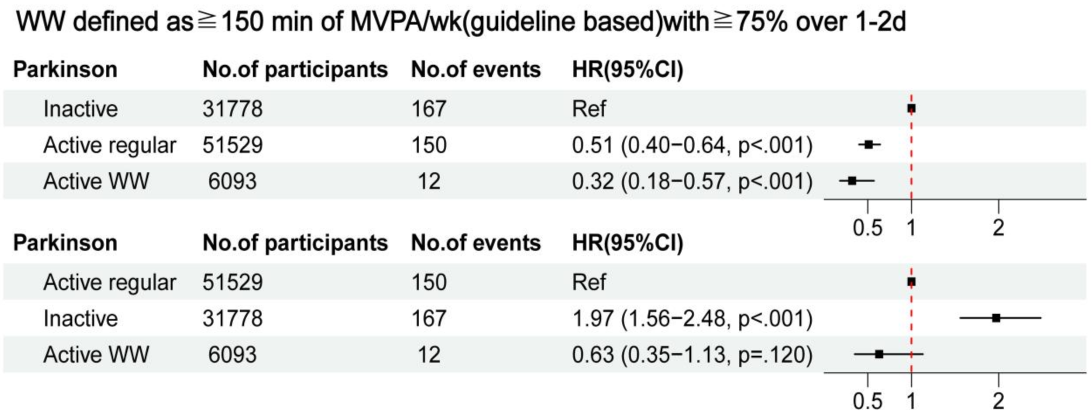

Supplementary Figure 4. Association between physical activity patterns and Parkinson's disease

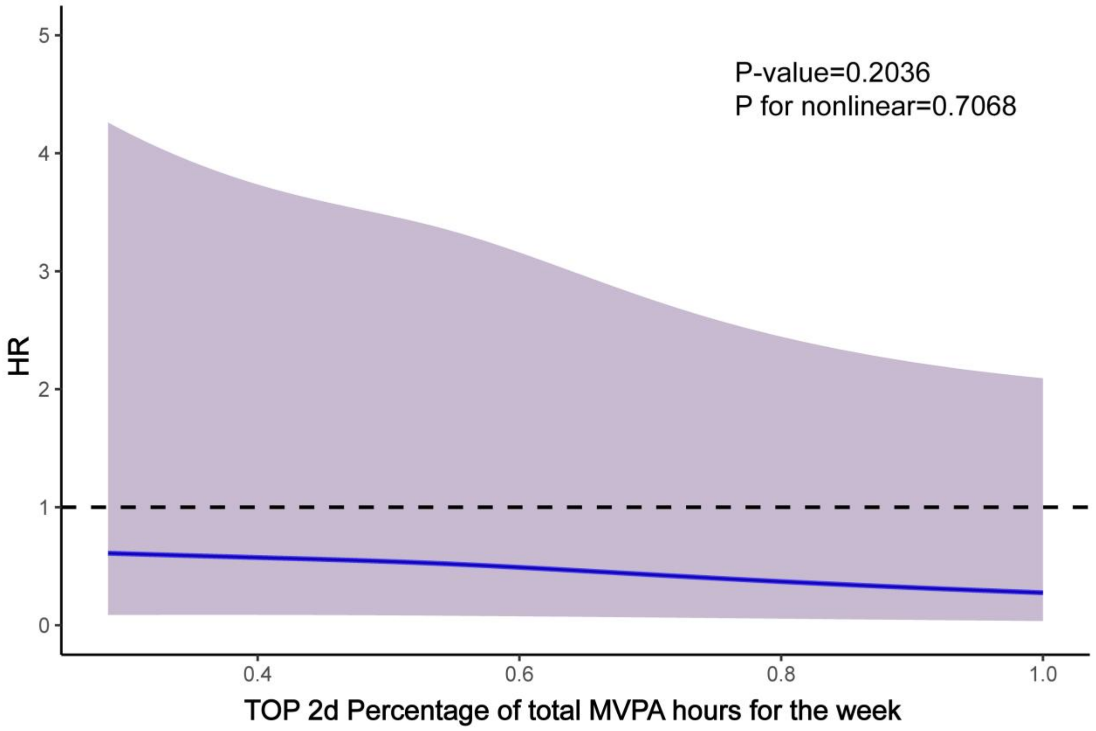

Supplementary Figure 5. Restricted cubic spline for testing the hypothesis of nonlinear correlation between TOP 2d Percentage of total MVPA hours for the week and the risk of PD.

The solid lines are fitted based on Cox-proportional hazard models. The shaded areas show 95% confidential intervals (CIs).

Variable	HR（95%CI，P-value）	P for interaction
**Sex**		0.1145
Female		
Inactive	Ref	
Active regular	0.32 (0.16-0.63, p=.001)	
Active WW	0.43 (0.27-0.68, p<.001)	
Male		
Inactive		
Active regular	0.69 (0.49-0.98, p=.038)	
Active WW	0.46 (0.33-0.62, p<.001)	
**alchol**		0.4532
Current		
Inactive		
Active regular	0.57 (0.42-0.78, p<.001)	
Active WW	0.44 (0.34-0.58, p<.001)	
Previous		
Inactive		
Active regular	0.32 (0.04-2.56, p=.285)	
Active WW	0.16 (0.02-1.24, p=.080)	
Never		
Inactive		
Active regular	0.87 (0.17-4.44, p=.865)	
Active WW	0.55 (0.12-2.55, p=.445)	
**hypertension group**		0.3475
Elevated		
Inactive		
Active regular	0.42 (0.18-1.01, p=.053)	
Active WW	0.30 (0.14-0.64, p=.002)	
Normal		
Inactive		
Active regular	0.99 (0.42-2.33, p=.976)	
Active WW	0.79 (0.36-1.71, p=.546)	
stage1		
Inactive		
Active regular	0.81 (0.46-1.42, p=.464)	
Active WW	0.49 (0.29-0.83, p=.008)	
stage2		
Inactive		
Active regular	0.46 (0.29-0.71, p=.001)	
Active WW	0.42 (0.29-0.60, p<.001)	
**Diabetes**		0.06578
No		

Inactive		
Active regular	0.57 (0.42-0.78, p<.001)	
Active WW	0.46 (0.35-0.60, p<.001)	
Yes		
Inactive		
Active regular	0.72 (0.24-2.18, p=.559)	
Active WW	0.09 (0.01-0.68, p=.020)	
**Family history of Parkinson's disease**		0.5407
No		
Inactive		
Active regular	0.61 (0.44-0.83, p=.002)	
Active WW	0.44 (0.33-0.58, p<.001)	
Yes		
Inactive		
Active regular	0.29 (0.10-0.91, p=.034)	
Active WW	0.46 (0.21-1.04, p=.061)	

Supplementary Table 1. Subgroup analysis of the association between participant movement patterns and Parkinson's disease

moderate to vigorous physical activity(MVPA)[1]	HR（95%CI，P-value）
Q1	
Active_regular	Ref
Active_WW	1.75 (0.43-7.08, p=.435)
Q2	
Active_regular	Ref
Active_WW	0.63 (0.36-1.10, p=.113)
Q3	
Active_regular	Ref
Active_WW	0.71 (0.43-1.19, p=.205)
Q4	
Active_regular	Ref
Active_WW	0.86 (0.51-1.47, p=.580)

Supplementary Table 2. Parkinson's risk between exercise modes after threshold cancellation

[1]Quartiles of moderate to vigorous physical activity. The cutoff points of the quartiles are101,230,403

TOP 2d Percentage of total MVPA hours for the week[1]	HR（95%CI，P-value）
Q1	Ref
Q2	1.05 (0.69-1.61, p=.816)
Q3	0.84 (0.54-1.30, p=.438)
Q4	0.80 (0.51-1.25, p=.321)
continue	0.34 (0.11-1.11, p=.075)

Supplementary Table 3. The effect of distribution of exercise intensity on Parkinson's risk

[1]Quartiles of TOP 2d Percentage of total MVPA hours for the week.The cutoff points of the quartiles are 0.456 , 0.538 , 0.643.
