# Supplementary material for: Association of physical activity pattern and risk of Parkinson’s disease
Source: NPJ Digit Med. 2024 May 23;7:137. doi: 10.1038/s41746-024-01135-3 (PMC11116521; doi:10.1038/s41746-024-01135-3)
Supplement: Supplementary file 2 — Reporting Summary [file 41746_2024_1135_MOESM2_ESM.pdf]

nature portfolio

Corresponding author(s): DAPR NPJDIGITALMED-09026

Last updated by author(s): Apr 24, 2024

Reporting Summary

Nature Portfolio wishes to improve the reproducibility of the work that we publish. This form provides structure for consistency and transparency in reporting. For further information on Nature Portfolio policies, see our Editorial Policies and the Editorial Policy Checklist.

Statistics

For all statistical analyses, confirm that the following items are present in the figure legend, table legend, main text, or Methods section.

n/a	Confirmed	
☐	☒	The exact sample size (*n*) for each experimental group/condition, given as a discrete number and unit of measurement
☐	☒	A statement on whether measurements were taken from distinct samples or whether the same sample was measured repeatedly
☐	☒	The statistical test(s) used AND whether they are one- or two-sided<br>*Only common tests should be described solely by name; describe more complex techniques in the Methods section.*
☐	☒	A description of all covariates tested
☐	☒	A description of any assumptions or corrections, such as tests of normality and adjustment for multiple comparisons
☐	☒	A full description of the statistical parameters including central tendency (e.g. means) or other basic estimates (e.g. regression coefficient) AND variation (e.g. standard deviation) or associated estimates of uncertainty (e.g. confidence intervals)
☐	☒	For null hypothesis testing, the test statistic (e.g. *F*, *t*, *r*) with confidence intervals, effect sizes, degrees of freedom and *P* value noted<br>*Give P values as exact values whenever suitable.*
☒	☐	For Bayesian analysis, information on the choice of priors and Markov chain Monte Carlo settings
☐	☒	For hierarchical and complex designs, identification of the appropriate level for tests and full reporting of outcomes
☒	☐	Estimates of effect sizes (e.g. Cohen's *d*, Pearson's *r*), indicating how they were calculated

Our web collection on statistics for biologists contains articles on many of the points above.

Software and code

Policy information about availability of computer code

Data collection	Data obtained from the UK Biobank are available on application at www.ukbiobank.ac.uk/register-apply (94166).
Data analysis	All statistical analyses in this study were performed using R software version 4.2.3.

For manuscripts utilizing custom algorithms or software that are central to the research but not yet described in published literature, software must be made available to editors and reviewers. We strongly encourage code deposition in a community repository (e.g. GitHub). See the Nature Portfolio guidelines for submitting code & software for further information.

Data

Policy information about availability of data

All manuscripts must include a data availability statement. This statement should provide the following information, where applicable:

- Accession codes, unique identifiers, or web links for publicly available datasets
- A description of any restrictions on data availability
- For clinical datasets or third party data, please ensure that the statement adheres to our policy

the UK Biobank data are available on application to the UK Biobank.

Research involving human participants, their data, or biological material

Policy information about studies with human participants or human data. See also policy information about sex, gender (identity/presentation), and sexual orientation and race, ethnicity and racism.

Reporting on sex and gender	The sex information is acquired from central registry at recruitment, but in some cases updated by the participant. Accelerometer-measured duration of moderate- to high-intensity exercise is associated with the risk of Parkinson's disease in the general population, and the effects of different exercise patterns on the risk of Parkinson's disease are similar. We also conducted subgroup analyses on sex and found that the findings apply to males and females.
Reporting on race, ethnicity, or other socially relevant groupings	The ethnicities information is acquired from central registry at recruitment through standardized questionnaires. Ethnicities were adjusted for in the multivariable model.
Population characteristics	This study included 89,400 individuals, with a mean follow-up period of 12.32 years. During the follow-up period, 329 participants developed PD. The MVPA data as defined by the Axivity AX3 wrist-worn triaxial accelerometer. The baseline table indicates that the active WW group had a higher proportion of men, higher income levels, increased education levels, lower Thomson Deprivation Index (TDI), fewer recent smokers, more recent drinkers, fewer patients with diabetes, and a higher weekly duration of MVPA compared with the inactive group. Participants in the active WW group shared identical study characteristics with those in the active regular group, except for a lower weekly MVPA duration in the active WW group (Table 1).
Recruitment	The UK Biobank is a large-scale cohort study conducted between 2006 and 2010, encompassing about 500,000 individuals aged 37–73 years. Initially, this cohort study recruited 502,389 individuals from the UK Biobank. The baseline data were collected at 22 study locations in England, Scotland, and Wales using touchscreen surveys, interviews, physical and functional assessments, as well as genetic and biological procedures.participants who provided a valid email address to UK Biobank were invited at random to wear a wrist-worn accelerometer (Axivity AX3). Finally this study utilized the inclusion of participants who had the original accelerometer dataset.
Ethics oversight	The study was approved by the North West Multi-centre Research Ethics Committee, the National Information Management Board, and the UK government. The ethical considerations for research involving individuals were thoroughly reviewed, and participants provided written informed consent.

Note that full information on the approval of the study protocol must also be provided in the manuscript.

Field-specific reporting

Please select the one below that is the best fit for your research. If you are not sure, read the appropriate sections before making your selection.

☒ Life sciences ☐ Behavioural & social sciences ☐ Ecological, evolutionary & environmental sciences

For a reference copy of the document with all sections, see nature.com/documents/nr-reporting-summary-flat.pdf

Life sciences study design

All studies must disclose on these points even when the disclosure is negative.

Sample size	89,400 participants
Data exclusions	Initially, this cohort study recruited 502,389 individuals from the UK Biobank. Further, 402,282 participants lacking information on daily and weekly MVPA exercise hours were excluded. Additionally, 100 participants with pre-existing PD were excluded. Finally, 10,607 participants with missing covariates, such as their health status and healthy eating scores, were excluded. Hence, the final study cohort comprised 89,400 individuals (Fig. 1).
Replication	This is a study based on data from the UK Biobank large-scale cohort. We have not repeated the analysis in other populations. However, we used different statistical methods to verify our findings: (1) controlling for a wide range of potential covariates; (2) comprehensive sensitivity analyses. Our main findings were quite robust and consistent across these analyses.
Randomization	This is an observational cohort study, and randomization is not applicable in this study
Blinding	This is an observational cohort study, and randomization is not applicable in this study.

Reporting for specific materials, systems and methods

We require information from authors about some types of materials, experimental systems and methods used in many studies. Here, indicate whether each material, system or method listed is relevant to your study. If you are not sure if a list item applies to your research, read the appropriate section before selecting a response.

Materials & experimental systems

n/a	Involved in the study
☒	☐ Antibodies
☒	☐ Eukaryotic cell lines
☒	☐ Palaeontology and archaeology
☒	☐ Animals and other organisms
☒	☐ Clinical data
☒	☐ Dual use research of concern
☒	☐ Plants

Methods

n/a	Involved in the study
☒	☐ ChIP-seq
☒	☐ Flow cytometry
☒	☐ MRI-based neuroimaging

Plants

Seed stocks	Not applicable
Novel plant genotypes	Not applicable
Authentication	Not applicable
